# Applications of a novel tumor-grading-metastasis staging system for pancreatic neuroendocrine tumors: An analysis of surgical patients from a Chinese institution

**DOI:** 10.1097/MD.0000000000004213

**Published:** 2016-07-18

**Authors:** Min Yang, Chun-Lu Tan, Yi Zhang, Neng-Wen Ke, Lin Zeng, Ang Li, Hao Zhang, Jun-Jie Xiong, Zi-Heng Guo, Bo-Le Tian, Xu-Bao Liu

**Affiliations:** a Department of Pancreatic Surgery; b General Ward of Sports Medicine & Cardiopulmonary Rehabilitation, West China Hospital of Sichuan University, Chengdu, Sichuan Province, the People's Republic of China.

**Keywords:** grading, pancreatic neuroendocrine tumors, staging, TGM, TNM

## Abstract

The ability to stratify patients with pancreatic neuroendocrine tumors (p-NETs) into prognostic groups has been hindered by the absence of a commonly accepted staging system. Both the 7th tumor-node-metastasis (TNM) staging guidelines by the American Joint Committee on Cancer (AJCC) and the 2010 grading classifications by the World Health Organization (WHO) were validated to be unsatisfactory.

We aim to evaluate the feasibility of combining the latest AJCC and WHO criteria to devise a novel tumor-grading-metastasis (TGM) staging system. We also sought to examine the stage-specific survival rates and the prognostic value of this new TGM system for p-NETs.

Data of 120 patients with surgical resection and histopathological diagnosis of p-NETs from January 2004 to February 2014 in our institution were retrospectively collected and analyzed. Based on the AJCC and WHO criteria, we replaced the stage N0 and N1 with stage *G*_*a*_ (NET G1 and NET G2) and *G*_*b*_ (NET G3 and MANEC) respectively, without changes of the definition of T or M stage. The present novel TGM staging system was grouped as follows: stage I was defined as T1–2, *G*_*a*_, M0; stage II as T3, *G*_*a*_, M0 or as T1–3, *G*_*b*_, M0; stage III as T4, *G*_*a*–*b*_, M0 and stage IV as any T, M1.

The new TGM staging system successfully distributed 55, 42, 12, and 11 eligible patients in stage I to IV, respectively. Differences of survival compared stage I with III and IV for patients with p-NETs were both statistically significant (*P* < 0.001), as well as those of stage II with III and IV (*P* < 0.001). Patients in stage I showed better a survival than those in stage II, whereas difference between stages III and IV was not notable (*P* = 0.001, *P* = 0.286, respectively). In multivariate models, when the TGM staging system was evaluated in place of the individual T, G, and M variables, this new criteria were proven to be an independent predictor of survival for surgically resected p-NETs (*P* < 0.05).

Stratifying patients well, the current proposed TGM staging system was predictive for overall survival of p-NETs and could be more widely applied in clinical practice.

## 1 Introduction

Deriving not only from mature pancreatic endocrine cells, but also from pluripotent stem cells of the pancreas,^[1]^ pancreatic neuroendocrine tumors (p-NETs) are considered to belong to amine precursor uptake and decarboxylation neoplasms, which may have the potentials to secrete some endocrine hormones, such as insulin, gastrin, glucagon, and so on.^[2]^ p-NETs are a heterogeneous group of malignancies with a common practice to label them as functional if patients have the symptoms of hormone overproduction, such as insulinoma with typical Whipple triad, and nonfunctional if patients are asymptomatic.^[3]^ Accounting for ∼3% of all pancreatic neoplasms,^[4]^ these uncommon p-NETs show an incidence of <5 cases per 1,000,000 persons each year.^[5]^ But their annual incidence has been increasing in the past years.^[6]^

Histopathological criteria for the diagnosis and classification of p-NETs have been widely established and validated in the current literatures to evaluate the biological behaviors of these unique tumors. However, due to their rarity and heterogeneity, the ability to risk-stratify patients and to provide prognostic information was hindered by the absence of an accepted staging system for p-NETs. Relying on the previous working efforts according to the clinicopathologic features of neuroendocrine tumors (e.g., tumor size, metastases, hormonal status, angiolymphatic invasion, mitotic rate, Ki-67 positive index, tumor differentiation, etc.),^[7]^ the World Health Organization (WHO) was the first one to introduce a system for both pathologic naming and classification of p-NETs in 2000.^[8]^ This WHO criteria were then updated and reclassified in 2010 into 4 main groups primarily referring to the Ki-67 labeling index and mitotic count: neuroendocrine tumor G1 (NET G1), neuroendocrine tumor G2 (NET G2), neuroendocrine carcinoma G3 (NEC G3), and mixed adeno and neuroendocrine carcinoma (MANEC).^[9]^ Moreover, the American Joint Committee on Cancer (AJCC) has been developing a tumor-node-metastasis (TNM) staging guidelines of solid tumors since the year of 1977. Nevertheless, it was not until 2010 that AJCC began to propose its TNM system for p-NETs (i.e., the 7th edition of AJCC staging manual).^[10]^ This system, however, was initially applied to the pancreatic exocrine adenocarcinoma, which also divided p-NETs into 4 stages distinguished between localized tumors (stage I), locally advanced but resectable tumors (stage II), locally advanced and unresectable tumors (stage III), and distantly metastasized tumors (stage IV).

The AJCC 2010 TNM staging system is prognostic for the survival of p-NETs, which has already been validated in some previous studies.^[11–16]^ However, this system has simultaneously been proven to show some drawbacks which limited its wider clinical use for p-NETs.^[13–16]^ For example, it does not consider histological grade or molecular subtypes such as mitosis and Ki-67 staging, though it recommends that tumor grade should be reported in conjunction with tumor stage. Also, compared with pancreatic exocrine adenocarcinoma, p-NETs have more indolent biological behaviors, which are more amenable to resection and have better long-term survival rates.^[17–18]^ Application of an identical AJCC staging manual for 2 different pancreatic diseases, although convenient, might be oversimplified. Thirdly, some studies have already demonstrated the predictive valve of lymph nodal status for p-NETs was limited and that the nodal stage showed no notable differences with respect to the estimated cumulative survival probability.^[19–24]^

On the other hand, the prognostic value of the newly updated WHO 2010 grading classifications has already been rigorously validated in our early-stage work.^[25,26]^ These WHO criteria made an important step toward defining the diverse biological features of p-NETs, which reflected the tumor's inherent malignant potential, whereas the AJCC system reflected the time of diagnosis or the progress of disease. Therefore, the different emphasis of these 2 systems for p-NETs might raise clinical concerns of potential confusions in patient management. With the expansion of annual incidence and surgical treatment of p-NETs, there is an obviously increasing need for all physicians to find a more applicable staging system of relevant prognostic factors which will be able to appropriately stratify these patients to determine better follow-up and additional therapy. Therefore, on the basis of both the AJCC 2010 staging manual and the WHO 2010 grading classifications, the objective of our present study was to evaluate the feasibility of combining these 2 classifications to devise a new tumor-grading-metastasis (TGM) staging system for p-NETs. In addition, compared with the AJCC criteria, we sought to examine stage-specific survival rates and the prognostic value for p-NETs using the new TGM system based on the data of all eligible patients in our single institution.

## 2 Materials and methods

### 2.1 Patient selection and tumor characteristics

This study enrolled a total of 120 consecutive patients from January 2004 to February 2014 in surgical departments of West China Hospital of Sichuan University. All patients were surgically treated and diagnosis of p-NETs was pathologically confirmed according to the histological analysis and immunohistochemical staining of surgical specimens or biopsy samples. Data, including patients’ demographics (gender and age), clinical presentations at admission (functional status), pathological analyses, surgical procedures, and in-hospital stays, and so on, were retrospectively collected from their electronic and/or paper-based medical records. Features of tumor (size, location, lymph invasion, distant metastasis, surgical margin, mitotic count, Ki-67 positive rate, etc.) were mainly referred to the intraoperative findings by surgeons and ultimate pathological analyses by pathologists of our hospital. All neoplasms were sporadic which originated only from pancreas. This research was approved by the local ethics committee, and written consent was provided for patient information to be used for research purposes.

### 2.2 Definitions of the AJCC staging, the WHO grading and the new TGM system

The newly updated WHO 2010 grading classifications were cited as follows: NET G1 (neuroendocrine tumor G1: mitotic count<2/10 high power fields[HPF], Ki-67<2%); NET G2 (neuroendocrine tumor G2: mitotic count: 2–20/10HPF, Ki-67: 3–20%); NEC G3 (neuroendocrine carcinoma G3: mitotic count>20/10HPF, Ki-67>20%); MANEC (mixed adeno-neuroendocrine carcinoma: 30% of either component required). The definitions of the AJCC 2010 TNM staging manual and the proposed novel TGM staging system were all listed in detail in Table 1. As we mentioned before, many studies have demonstrated the predictive valve of lymph nodal status for the survival analysis of p-NETs was limited,^[19–24]^ whereas the WHO 2010 grading classifications were proven to present notable prognostic significance.^[25,26]^ Considering the unique biological behaviors of p-NETs and combining the current WHO and AJCC criteria, we designed in the present study to replace the stage N0 and N1 with stage *G*_*a*_ and *G*_*b*_, respectively, in order to attempt to remedy the shortcomings of the AJCC TNM system and to devise the novel TGM staging system. Of those, *G*_*a*_ was composed of NET G1 and NET G2, whereas *G*_*b*_ was made up of NET G3 and MANEC according to the new WHO 2010 grading classifications. Meanwhile, in accordance with the AJCC 2010 staging manual, we did not change the definition of T or M stage when forming the new one. Thus, the new TGM staging system was determined as follows: stage I was defined as T1–2, *G*_*a*_, M0; stage II as T3, *G*_*a*_, M0 or as T1–3, *G*_*b*_, M0; stage III as T4, *G*_*a*–*b*_, M0 and stage IV as any T, M1. The new WHO grading classifications, the AJCC 2010 staging manual, and the present TGM staging system were all applied wherever possible in this study.

**Table 1:**
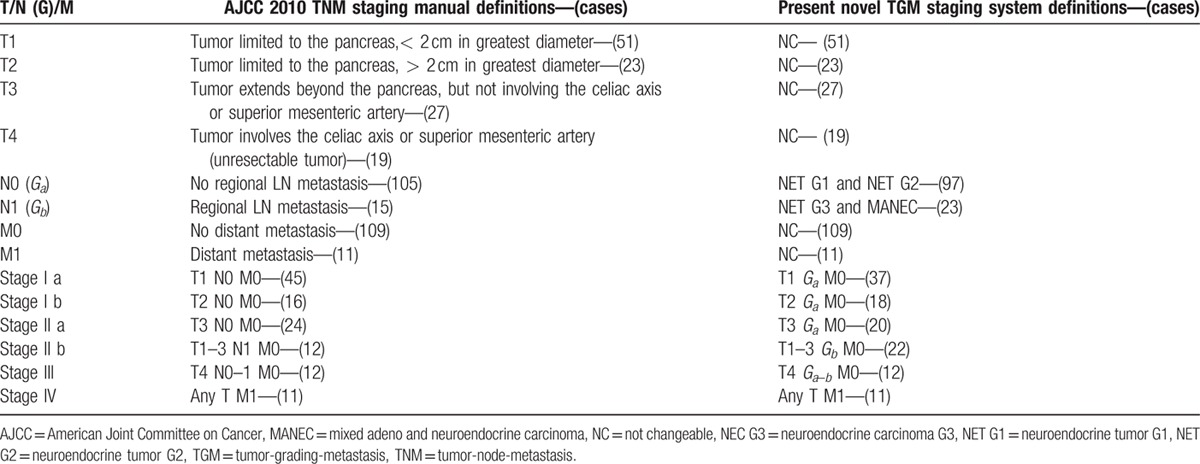
The original definitions and current analyses of 2 staging criteria.

### 2.3 Survivals and statistical analyses

Follow-up was conducted from August to October 2014 by telephone, office visit, and outpatient clinic. Patients who were lost to follow-up were not enrolled in this study. Overall Survival (OS) was defined as the number of months from the date of resection to the time of death or last contact. Data were presented as mean ± standard error of mean (SEM) or median for quantitative variables, or as numbers and their frequencies with proportions (%) for categorical variables unless otherwise indicated. Kaplan–Meier curves were plot and log-rank test were performed to analyze and compare the OS. Univariate and multivariate analyses were also applied to evaluate the prognostic value of related factors by Cox Regression proportional hazards model. *P* value of 2 sides <0.05 was considered statistically significant. Data analyses were performed by IBM SPSS17.0 statistical software.

## 3 Results

From January 2004 to February 2014 in our hospital, a gross of 120 patients with surgical resections who were all histologically diagnosed as p-NETs were identified in our series. Relevant clinical–pathological characteristics of all subjects were summarized in Table 2.^[26]^ Our analyses consist of 50 males (41.7%) and 70 females (58.3%), with a median age at initial diagnosis of 47 years (ranging from 14 years to 77 years). The tumor diameters varied from 0.3 cm to 12 cm, with a median of 2 cm. Tumors were located in the head and uncinate of pancreas in 53 patients (44.2%), body and tail in 67 cases (55.8%). Contrast to many studies in the Europe or United States, the most common diagnosis of p-NETs was functional ones (87, 72.5%), in which 80 patients were both clinically and pathologically diagnosed as insulinoma (66.7%), whereas only 33 patients (27.5%) did not manifest the symptoms related to hormone overproduction. As for the WHO 2010 grading classifications, 62 patients were histologically diagnosed as NET G1 (51.7%), 35 NET G2 (29.2%), 17 NET G3 (14.1%), and 6 MANEC (5.0%). Fifteen patients were pathologically confirmed to have lymph node invasion (12.5%), whereas 11 cases present distant metastases (9.2%). Surgical treatments were performed for all patients, in which 106 patients underwent radical resection (i.e., surgical margin was immunohistochemically negative for the tumor tissue) (88.3%), whereas 14 cases underwent only palliative operation due to the unresectable tumors (11.7%). When the follow-up ended in October 2014, 86 patients were still alive (71.7%) whereas 34 ones were dead related to the tumor progression (28.3%), with a death rate of 28.3%.

**Table 2:**
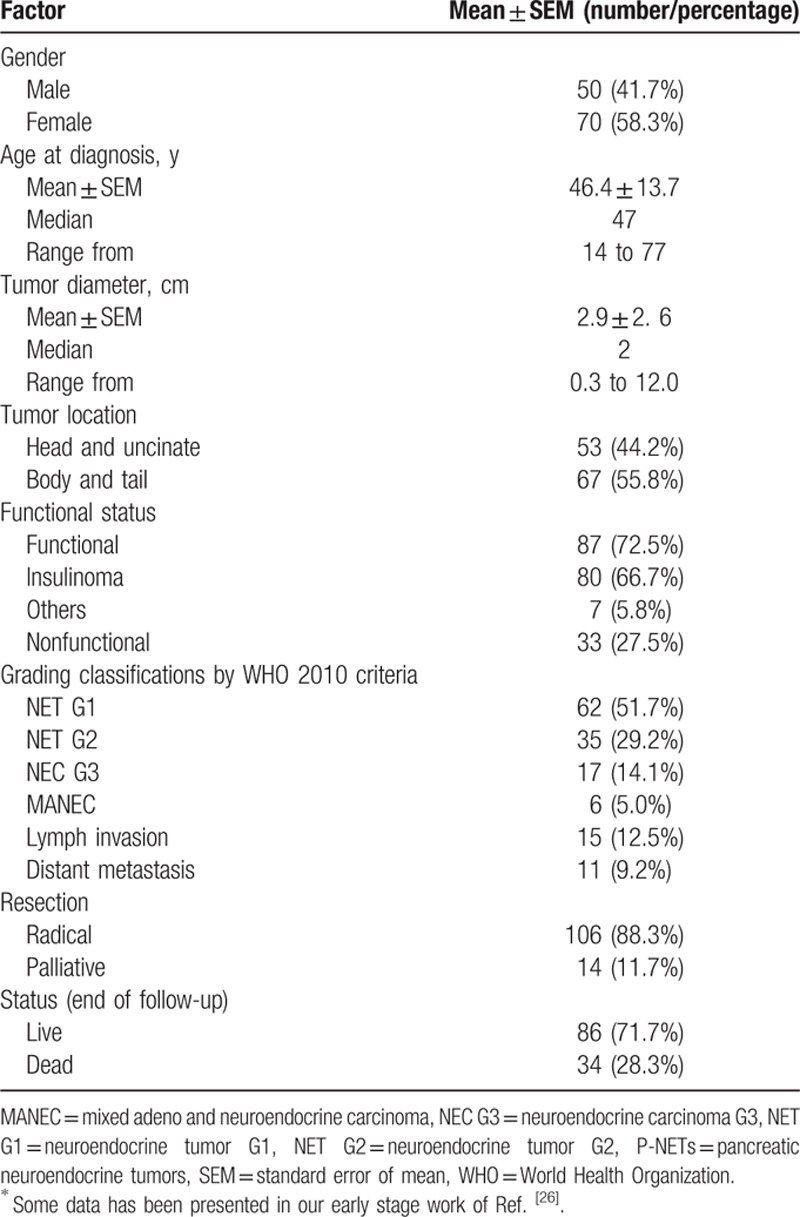
Demographics baseline and tumor features of p-NETs in the present study^∗^.

As shown in Table 1, the present new TGM staging system expectedly assigned each patient into different stages. For we did not change the definition of T or M stage when forming the new one, the stage from T1 to T4 were distributed respectively to 51, 23, 27, and 19 patients, which were consistent with those by AJCC criteria. Also, there were 11 patients in the M1 stage. As we defined before for the new system, 97 patients were assigned in stage *G*_*a*_ (80.8%), whereas stage *G*_*b*_ was grouped with 23 cases (19.2%). Finally, in terms of the present novel TGM staging system, we devised stage I, II, III, and IV were distributed in 55, 42, 12, and 11 patients, respectively.

**Figure 1:**
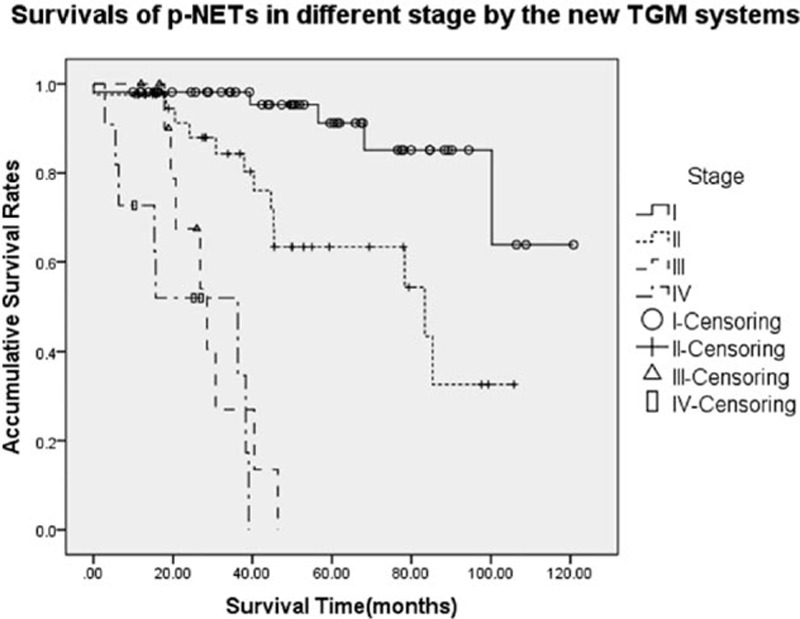
Survivals of p-NETs in different stages by the present proposed TGM staging system. Differences of stage I or II with stage III or IV were both significant (*P* < 0.001). Survival of p-NETs in stage I was statistically better than that in stage II, whereas that compared stage III with IV was not notable (*P* = 0.001, *P* = 0.286, respectively). p-NETs = pancreatic neuroendocrine tumors, TGM = tumor-grading-metastasis.

The survival analysis by Kaplan–Meier curves calculated the 3-year OS of the entire cohort by the new TGM system from stage I to IV was 98.2%, 84.3%, 27.0%, and 34.6%, respectively, whereas OS at 5 years was respectively 91.2%, 63.4%, NA (not applicable), NA (*P* < 0.001, Fig. 1). The median survival time (MST) of the TGM stage I to IV was NA, 83.4, 28.6 and 36.3 months, respectively. Detailedly about the TGM staging system, differences of survival for patients with p-NETs compared stage I with stages III and IV were both significant (*P* < 0.001, *P* < 0.001, respectively), as well as those of stage II with stages III and IV (*P* < 0.001, *P* < 0.001, respectively). Furthermore, survival of p-NETs in stage I was statistically better than that in stage II (*P* = 0.001). Although the MST of stage III was a little shorter compared with that of stage IV, their difference was not notable (28.6 vs 36.3 months, *P* = 0.286).

**Figure 2:**
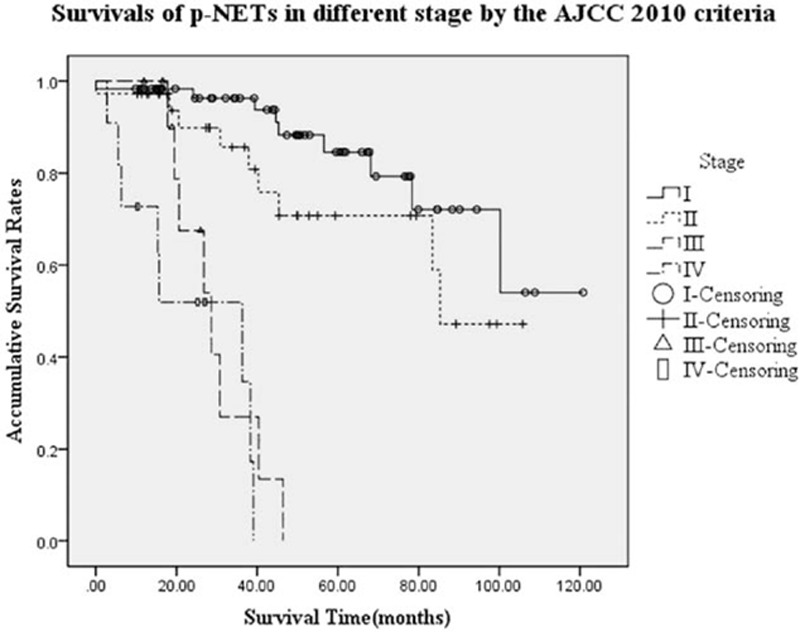
Survivals of p-NETs in different stages by the AJCC 7th staging manual.^[26]^ Differences of stage I or II with stage III or IV were also both notable (*P* < 0.001), whereas comparisons of stage I with stage II and stage III with IV were not significant (*P* = 0.129, *P* = 0.286, respectively). AJCC = American Joint Committee on Cancer, p-NETs = pancreatic neuroendocrine tumors.

**Figure 3:**
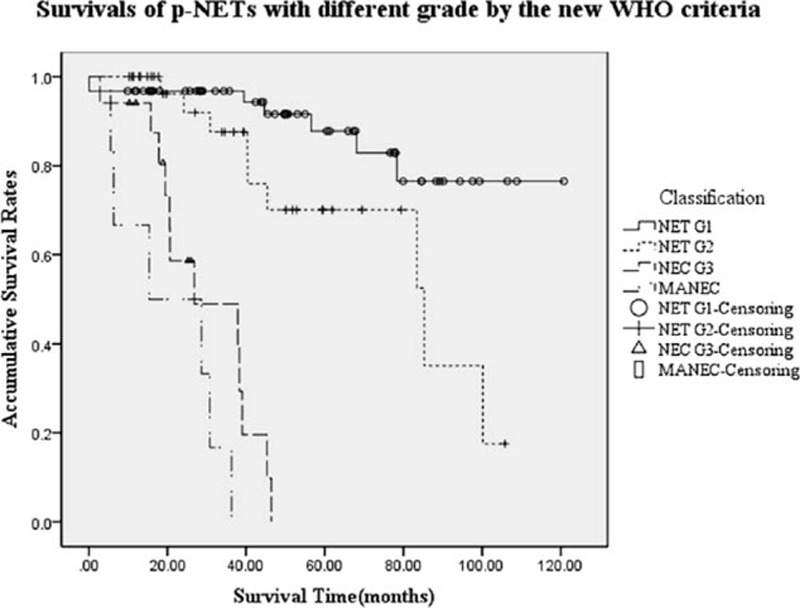
Survivals of p-NETs with different grades by the new WHO 2010 grading classifications.^[26]^ Patients with NET G1 or NET G2 both showed a better survival compared with those with NEC G3 or MANEC (*P* < 0.001). Survivals of NET G1 was longer than those of NET G2 (*P* = 0.023), whereas difference of survivals between NEC G3 and MANEC present no obvious significance (*P* = 0.071). MANEC = mixed adeno and neuroendocrine carcinoma, NEC G3 = neuroendocrine carcinoma G3, p-NETs = pancreatic neuroendocrine tumors, WHO = World Health Organization.

As we reported in our early-stage work,^[26]^ the AJCC 2010 TNM staging system was also accordingly applied to all subjects in the present study, with a distribution of 61, 36, 12, and 11 patients for each stage. The OS rate at 5 and 3 years for these criteria stage I to IV were 84.6%, 70.7%, NA, NA and 96.3%, 85.6%, 27.0%, 34.6%, respectively (*P* < 0.001, Fig. 2). Although differences of survival of stage I with stage III and IV were also similarly significant (*P* < 0.001, *P* < 0.001, respectively), as well as those compared stage II with stage III and IV (*P* < 0.001, *P* < 0.001, respectively), comparisons of stage I with II or stage III with IV both did not present any notable difference (*P* = 0.129, *P* = 0.286, respectively). As for the tumor differentiations by WHO 2010 grading classification, similar results could be seen in Fig. 3.

Our previous study has also concluded that patient's gender, age, tumor dimension, location, and functional status were not predictive for the survival analysis of p-NETs, whereas radical resection, stages by AJCC 7th manual and gradings by WHO 2010 criteria were all statistically prognostic factors.^[26]^ In the present study, moreover, to evaluate the independent effects of T, N, G, and M stage on survival of resected p-NETs, multivariate models were also conducted by controlling and adjusting for patient gender, age, tumor location, and functional status (Table 3). We validated T stage did not show any significant effect on survival (*P* > 0.05) and that nodal invasion were also not an independent prognostic factor (*P* = 0.285). However, both distant metastasis and tumor grading by WHO 2010 classifications were associated with a growing likelihood of death (*P* < 0.001, *P* = 0.028, respectively). Meanwhile, exactly as we demonstrated before, the AJCC 2010 TNM staging manual was once again confirmed to own its prognostic value for p-NETs (*P* < 0.05). When the present TGM staging system was evaluated in place of the individual T, G, and M parameters, this new system was also an independent predictor of survival for surgically resected p-NETs (*P* < 0.05).

**Table 3:**
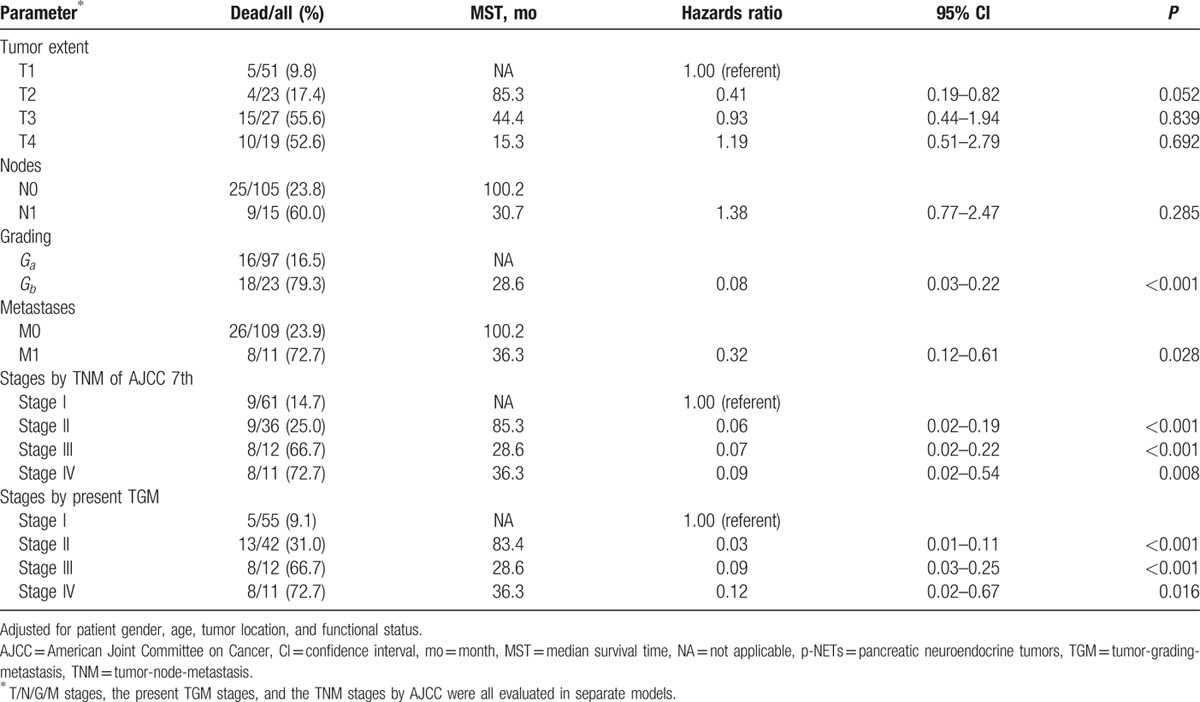
Multivariate analysis for the independent effects of T/N/G/M stages, the present new TGM staging system, and the TNM stages by AJCC 7th on survival after resection of p-NETs.

## 4 Discussion

p-NETs, namely islet cell tumors, are a heterogeneous group of neoplasm with a wide spectrum of biological behaviors from benign to malignant.^[6]^ Currently, due to its rarity and heterogeneity, p-NETs have not been well studied as pancreatic adenocarcinoma^[27]^ and a widely accepted staging system for p-NETs has always been absent. As we all know, the AJCC has been developing a TNM staging guidelines of solid tumors since 1977, but it in 2002 (i.e., AJCC 6th edition) still excluded p-NETs when staging pancreatic tumors as they ever did.^[28]^ Nevertheless, the AJCC 2010 staging manual (i.e., AJCC 7th edition) first introduced its TNM staging system to p-NETs, although it originally derived from the staging algorithm for pancreatic adenocarcinomas.^[10]^ Obviously, this was an important step toward adopting a uniform staging system for p-NETs, which was soon endorsed by other great international organizations.^[29,30]^

In 2011, Strosberg et al^[11]^ successfully evaluated the clinical value of the AJCC 2010 staging manual for p-NETs for the first time. They concluded the TNM system of the AJCC 7th edition was prognostic for OS rates of p-NETs and that it could be adopted in clinical practice, which was also widely validated in some subsequent researches.^[12–16,26]^ However, the AJCC staging manual was originally applied to pancreatic exocrine adenocarcinomas, whose long-term survival was much worse than that of p-NETs. Therefore, it might be oversimplified to use the same criteria for 2 different diseases. In 2012, Rindi et al^[31]^ first reported the AJCC 2010 TNM staging system only compressed p-NETs into 3 differently populated classes, with most patients in stage I, and with the patients being equally distributed into stages II–III (statistically similar) and IV (*P* < 0.001). They concluded that the AJCC 7th manual might not be the most suitable and practical staging system for the survival analysis of p-NETs. Furthermore, in 2014, based on the eligible data of 412 patients, Qadan et al^[13]^ demonstrated the current AJCC staging system distinguished 5-year OS only between stage I and II (84% vs 72%; *P* = 0.01), but not between stage II and III (72% vs 65%; *P* = 0.97), or stage III and IV (65% vs 55%; *P* = 0.36). They then proposed that a revised TNM staging system which could better discriminate the outcomes of surgically resected p-NETs should be considered. Similar with our previous studying effort,^[15,26]^ the 5-year OS rate by AJCC criteria stage I to IV in the present study were 84.6%, 70.7%, NA, NA (*P* < 0.001). Although we succeeded in stratifying all patients into 4 stages, differences of survival of stage I with stage III and IV were statistically significant (*P* < 0.001), as well as those of stage II with stage III and IV (*P* < 0.001), whereas comparisons of stage I with II or stage III with IV did not present any notable difference (*P* = 0.129, *P* = 0.286, respectively). It could be seen in Fig. 2 that the survival curves of stage I with II, and stage III with IV intertwined tightly with each other.

Meanwhile, accumulative studies have already demonstrated that the N stage showed no notable differences with respect to the estimated cumulative survivals of p-NETs and concluded that the predictive valve of lymph nodal status was limited,^[20–24]^ which agreed with what we reported before.^[19]^ When Bilimoria et al^[32]^ at the earliest tried and succeed to apply the AJCC 6th staging manual into p-NETs, they also reported that lymph nodal was not independent predicting factor (*P* = 0.62), whereas only distant metastasis was the significant one (*P* < 0.0001). Their conclusion were analogously validated in some later series using the AJCC 7th TNM staging system.^[11–13,15,31]^ In the present study, we calculated in the multivariate analysis the similar results with the significant factor of distant metastases and the meaningless one of lymph nodal (*P* = 0.028, *P* = 0.285, respectively). So, as the traditional prognostic factor of outcome of many solid tumors, nodal status was not accurate and powerful predictor to define the heterogeneous biological behaviors of p-NETs.

On the other hand, on the basis of many existing efforts,^[7,8]^ the WHO recently updated and reclassified its system for p-NETs (i.e., the WHO 2010 grading classifications).^[9]^ This system accurately recognized the clinical, molecular, and histopathologic characteristics of p-NETs, which would be an effective scheme and a clear guideline to assist clinicians in the patients’ management of p-NETs. Our 2 early stage work have validated the clinical and prognostic value of this new WHO system,^[25,26]^ which was once again confirmed in the present analysis (*G*_*a*_ vs *G*_*b*_; *P* < 0.001). Like we said before, the WHO grading criteria made an important step toward defining the diverse biological features of p-NETs which reflected the tumor's inherent malignant potential, whereas the AJCC TNM system reflected the time of diagnosis or the progress of disease. Therefore, our original intention was to devise a TGM staging system that could take into considerations both the T and M stage of AJCC 7th staging manual, in combination with the G stage of WHO 2010 grading criteria. We hope this novel TGM system would effectively remedy the drawbacks of both the AJCC and WHO criteria and that it be more easily accepted and more widely applied for better follow-up and additional therapy of p-NETs.

Our present study was the first attempt to successfully integrate the AJCC 2010 TNM staging system with the WHO 2010 grading classifications. Actually, we found that there were good stage-specific survival discriminations for p-NETs through the new TGM staging system. We thoroughly assigned all 120 eligible patients into stage Ito IV. Besides the significant differences of stage I or II with stage III or IV (*P* < 0.05), we also detected that patients in TGM stage I showed a statistically better survival than those in stage II, whereas the AJCC staging system failed to distinguish between stage I and II (*P* = 0.001, *P* = 0.129, respectively). Interestingly, differences between stages III and IV were not notable by both the AJCC criteria and the present system (both *P* = 0.286). This distinction could probably be explained by the definitions of the TGM system: we respectively replaced the stage N0 and N1 with stage *G*_*a*_ (*G*_*a*_ = NET G1 and NET G2) and *G*_*b*_ (*G*_*b*_ = NET G3 and MANEC), which has been proven to be significant predictor of p-NETs in the multivariate analysis (*P* < 0.001), like we reported before.^[25,26]^ Meanwhile, consistent with the AJCC manual, we did not change the definition of T or M stage (Table 1). These differences led to the redistribution of stages I and II (n = 55, n = 42, respectively), whereas quantity of patients in stages III and IV was changeless (n = 12, n = 11, respectively), compared with those by AJCC criteria with assignments of 61, 36, 12, and 11 patients for each stage. Then, patients in stage III (locally advanced and unresectable tumors) hereby showed a little shorter MST than those in stage IV (distantly metastasized tumors), although the difference was not notable (28.6 vs 36.3 months, *P* = 0.286). This was probably because patients with p-NETs could benefit from surgical treatments if radical resection of the primary tumor was achieved, even for those with distant metastatic lesions.^[5]^ At last, we still confirmed the present TGM staging system was an independent survival predictor for surgically resected p-NETs, which was examined in place of the individual T, G, and M variables (*P* < 0.05). Our analysis demonstrated the clinical and prognostic value of the present TGM staging system for the outcome of p-NETs, which might provide us the promising theoretical foundations for its wider clinical use.

Our study had some limitations as well, the major of which was its retrospective nature with the potential error and variation when collecting information, such as the tumor histopathologic features and patients follow-up. Also, all patients were surgically treated and diagnosed as p-NETs histologically, either radical or palliative resections. Patients with only clinical suspicion but not postoperatively pathological confirmations were not enrolled in this study, which was inevitable to miss some cases that did not undergo a surgery. Then, unlike many studies abroad, the most common subgroup of p-NETs in the present cohort was functional (87, 72.5%), in which insulinoma accounted most (80, 66.7%), whereas only 33 patients (27.5%) were nonfunctional. This might result to an increasing error when using the AJCC staging manual. Finally, the new TGM staging system also failed to distinguish the OS rate between stages III and IV (*P* = 0.286), which meant any in-depth evaluations and more improvements of the present staging system or other new classifications are still needed to be further researched.

## 5 Conclusion

In a word, we successfully made the first try to integrate the AJCC 7th TNM staging manual with the WHO 2010 grading classifications. Our data indicated that applying the present novel TGM staging system for the survival analysis of surgically resected p-NETs was appropriate and promising. We also succeed in examining stage-specific survival rates and validating the prognostic value of this new system for p-NETs, which might be superior to the simple AJCC 2010 criteria. Applications of this newly devised TGM staging system into clinical practice would enhance the ability to risk-stratify patients and predict prognosis of p-NETs.
